# Reduced induction of anti-PF4/heparin antibody in RA patients after total knee arthroplasty

**DOI:** 10.1186/s13075-016-1090-2

**Published:** 2016-08-25

**Authors:** Masahiro Izumi, Tatsuya Sakai, Atsunori Shirakawa, Hideko Kozuru, Yuka Jiuchi, Yasumori Izumi, Tomohiko Asahara, Kenji Kumagai, Masaaki Mawatari, Makoto Osaki, Satoru Motokawa, Kiyoshi Migita

**Affiliations:** 1 Department of Molecular Immunology, Unit of Hepatology, Nagasaki University Graduate School of Biomedical Sciences, Sakamoto 1-7-1, Nagasaki, 852-8501 Japan; 2 Department of Orthopedic Surgery, NHO Nagasaki Medical Center, Kubara 2-1001-1, Omura, Nagasaki 856-8562 Japan; 3 Department of Pharmacy, NHO Nagasaki Medical Center, Kubara 2-1001-1, Omura, Nagasaki 856-8562 Japan; 4 Department of Rheumatology, Clinical Research Center, NHO Nagasaki Medical Center, Kubara 2-1001-1, Omura, Nagasaki 856-8562 Japan; 5 Department of Orthopedic Surgery, Saga University Hospital, Nabeshima 5-1-1, Saga, 849-8501 Japan; 6 Department of Orthopedic Surgery, Nagasaki University Hospital, Sakamoto 1-7-1, Nagasaki, 852-8501 Japan; 7 Suga Orthopedic Hospital, Ono 332, Isahaya, 854-0034 Japan; 8 Department of Rheumatology, Fukushima Medical University School of Medicine, Hikarigaoka 1, Fukushima, Fukushima 960-1295 Japan

**Keywords:** Heparin-induced thrombocytopenia, Platelet factor 4, Rheumatoid arthritis, Osteoarthritis, Total knee arthroplasty, Anti-PF4/heparin antibodies

## Abstract

**Background:**

Heparin-induced thrombocytopenia is caused by antibodies (Abs) specific to platelet factor 4 (PF4)/heparin complexes. In this study, we evaluated the rates of seroconversion of anti-PF4/heparin Ab between patients with rheumatoid arthritis (RA) and with osteoarthritis (OA) who underwent total knee arthroplasty.

**Methods:**

The subjects of this randomized controlled trial were 124 patients who underwent total knee arthroplasty (TKA) and received edoxaban with or without a foot pump as thromboprophylaxis. We measured anti-PF4/heparin Abs before and 10 days after surgery, as well as preoperative PF4, using commercially available ELISAs. We also used the database of J-PSVT, a hospital-based, prospective cohort study designed to document the effectiveness of thromboprophylactic agents during arthroplasty.

**Results:**

The rates of seroconversion to anti-PF4/heparin Ab were lower in RA patients (4.0 %) than in OA patients (25.5 %). The anti-PF4/heparin IgG optical density (OD) values did not differ before and after surgery in RA patients. In contrast, there was a significant increase in anti-PF4/heparin IgG OD values in OA patients after TKA. In the J-PSVT data, the postoperative seroconversion rates of anti-PF4/heparin Ab were lower in RA patients (10.4 %) than in OA patients (21.8 %) who received fondaparinux. The titers of anti-CCP Ab were significantly lower in RA patients with postoperative ant-PF4/heparin Ab compared with those without postoperative ant-PF4/heparin Ab There was no significant difference in preoperative PF4 levels between RA patients and OA patients. The heparin-binding affinity of the circulating PF4 was similar between RA patients and OA patients; however, the IgG fractions isolated from the sera of RA patients contained PF4 more frequently (69.2 %) than those from OA patients (10.2 %).

**Conclusions:**

Our results showed a reduced likelihood of postoperative anti-PF/heparin Ab production in RA patients compared with OA patients. This suggests that the mechanisms underlying the anti-PF4 immune response in RA patients differ from the mechanisms of the anti-PF4/heparin immune response seen in OA patients after joint replacement.

**Trial registration:**

ISRCTN 18090286. Registered 8 July 2016

## Background

Heparin-induced thrombocytopenia (HIT) is an immune-mediated disorder that can develop during anticoagulant therapy with heparin [1]. HIT is associated with antibodies (Abs) that recognize the complexes formed between platelet factor 4 (PF4) and heparin [2]. There is increasing evidence that immune complexes formed by IgG Abs and the PF4/heparin complex bind and activate platelets resulting in acceleration of the coagulation pathway [3]. Recently, anti-PF4/heparin Abs have also been demonstrated in individuals who have not received heparin treatment [4]. PF4 could be an antigenic target in autoimmune diseases [5]. Additionally, anti-PF4/heparin Abs can be induced in patients who have undergone major surgery, even without any exposure of unfractionated heparin [6]. The development of HIT has been associated with postoperative thromboprophylaxis with fondaparinux, a factor Xa inhibitor [7]. Other examples of so-called “spontaneous” HIT preceding inflammatory or infectious triggers have been reported [8]. Krauel et al. [9] proposed that bacterial infections could trigger anti-PF4/heparin Ab formation. Anti-PF4/heparin IgG Abs are generated in approximately 20 % of patients undergoing total knee arthroplasty (TKA) [10]. In addition to the administration of heparin for anticoagulation during thromboprophylaxis, TKA poses a substantial challenge to the immune system because of mechanical damage to articular connective tissue [11].

PF4 is rapidly released after platelet activation and forms tetramers that bind to polyanions such as heparin, forming PF4/heparin complexes [12]. The resultant immune complexes induce the immune response resulting in the production of anti-PF4/heparin Abs [13]. However, the immunobiology of the anti-PF4/heparin Ab response is not well understood. Among surgical procedures, joint replacements result in extremely high rates of postoperative anti-PF4 Abs. The joint replacement procedure itself might be capable of triggering anti-PF4 Ab formation in both patients with osteoarthritis (OA) and those with rheumatoid arthritis (RA), which is an autoimmune disease; however, the frequency of and risks associated with anti-PF4/heparin Ab formation in RA patients who undergo joint replacement surgery without heparin exposure remain unclear.

In this study we compared the rate of seroconversion with anti-PF4/heparin Ab positive status between patients with OA and with RA during the postoperative period after TKA.

## Methods

### Study subjects

Between September 2013 and March 2015 a total of 120 patients were enrolled in this randomized controlled trial. Patients were given edoxaban alone or edoxaban plus a foot pump as described previously [14]. The primary effectiveness outcome was total venous thromboembolisms (VTEs); these included asymptomatic deep vein thromboses (DVTs) up to postoperative day (POD) 10, symptomatic DVTs, and fatal/nonfatal pulmonary embolisms (PEs) up to POD 28. The study protocol was approved by the ethics committees at the Nagasaki Medical Center (Protocol No. 25004), and all patients gave their written informed consent to participate. This study was registered in the ISRCTN registry (ISRCTN 18090286).

The Japanese Study of Prevention and Actual Situation of Venous Thromboembolism after Total Arthroplasty (J-PSVT) is a hospital-based, prospective cohort study designed to document the effectiveness and safety of current standard thromboprophylactic agents, including unfractionated heparin (UFH), low-molecular-weight heparin (LMWH), fondaparinux, and antiplatelet agents, approved for use in Japan [15]. Data were collected prospectively on all patients undergoing primary TKA and total hip arthroplasty (THA) in 34 NHO hospitals since 2007–2010. The primary aim of the J-PSVT was to determine the rates of VTEs and seroconversion rates of anti-PF4/heparin Ab in patients undergoing TKA and THA, with all patients evaluated for the presence of all (symptomatic/nonsymptomatic) DVTs on POD 10. The trial was registered in the Japan UMIN Clinical Trial Registry (UMIN000001366). The study protocol was approved by the ethics committees of the National Hospital Organization central institutional review board (No 0623004). Written informed consent was obtained from each individual for their clinical records to be used in this study.

### Blood sampling

Serum samples were obtained before surgery and at POD 10 and were stored at −30 °C. An enzyme-linked immunosorbent assay (ELISA) kit (GTI Diagnostics, Waukesha, WI, USA) was used to measure the IgG-class anti-PF4/heparin Ab (HIT Ab) according to the manufacturer’s instructions. ELISA reactivity (optical density (OD)) was expressed relative to a standard control. The cutoff value was set at 0.50 OD. We defined seroconversion as a negative ELISA result before surgery followed by a positive result on POD 10 with a 2-fold or more increase in OD, as defined in a previous study [14]. HIT is characterized by a decrease in the platelet count 50 % beginning 5–10 days after the start of heparin or major surgery in association with the appearance of platelet-activating anti-PF4/heparin Ab [16]. We therefore analyzed the anti-PF4/heparin Ab at POD 10.

In J-PSVT, anti-PF4/heparin Abs were evaluated using a commercially available ELISA for detecting IgG, IgA, and IgM (HPIA; Diagnostica Stago, Asnières-sur-Seine, France) at a central laboratory by personnel blinded to all clinical information. The cutoff value was approximately 0.4–0.5 OD units, depending on the assay kit. Each kit has a specific reference standard to determine the cutoff value. We defined seroconversion as a positive test result on POD 10 corresponding to a negative result before surgery. In addition, we considered patients who tested positive before surgery to be seroconverters if their blood sample on POD 10 was positive with a 2-fold or higher increase in OD.

### Immunoblot analysis

Serum samples (8 μl) were diluted 10-fold with phosphate-buffered saline (PBS) and incubated with protein G-Sepharose-4B beads or heparin-Sepharose-4B beads (Invitrogen Carlsbad, CA, USA) for 30 min with gentle mixing. The beads were washed with PBS three times and eluted with 20 μl of lithium dodecyl sulfate (LDS) sample buffer at 70 °C for 10 min. The eluted protein fractions were then separated under reducing conditions by NuPAGE 3–8 % Tris-acetate gel electrophoresis (Invitrogen). Proteins were electrophoretically transferred onto an Invitrogen polyvinylidene fluoride membrane and incubated overnight at 4 °C with blocking solution (5 % nonfat milk in Tris-buffered saline with 0.05 % Tween 20 (TTBS)). The blocked membrane was incubated with rabbit anti-human PF4 polyclonal antibody (Abcam, Cambridge, UK; 1:10,000 dilution with 1 % nonfat milk in TTBS) for 1 h at room temperature and then washed five times with TTBS buffer for 10 min each time at room temperature with constant shaking. The membrane was then incubated with horseradish peroxidase-conjugated second antibody (1:3,000 dilution; Santa Cruz Biotechnology) for 1 h at room temperature and washed five times with TTBS buffer for 10 min each time at room temperature with constant shaking. Immunodetection was performed using an enhanced chemiluminescence western blotting kit (Amersham, Little Chalfont, UK). Sera from 13 healthy subjects (six males, seven females; mean age of 42.7 ± 12.7 years) were used as controls.

### Measurement of serum PF4

Serum PF4 concentrations were assayed using an ELISA kit from R&D Systems (Minneapolis, MN, USA) according to the manufacturer’s instructions.

### Statistical analysis

The significance of differences in discrete and continuous variables was assessed using chi-square and Mann–Whitney tests, respectively. Multivariate logistic regression was performed to identify independent risk factors for anti-PF4/heparin Ab seroconversion. Variables with *p* < 0.25 by chi-square test or Fisher’s exact test in the univariate logistic regression analysis were selected for the multivariate model. The Wilcoxon signed rank test was used for the comparison of a pair of samples. All data processing and analyses were performed using the Statistical Analysis System (SAS) and SPSS version 18 (SPSS, Chicago, IL, USA).

## Results

### Patient demographic data

The characteristics of the total study population that underwent TKA are presented in Table 1.

**Table 1:** Baseline characteristics of RA and OA patients

	Total subjects	RA patients	OA patients	
	(*n* = 124)	(*n* = 26)	(*n* = 98)	*p* value
Gender (male/female)	20/104	2/24	18/80	0.188
Age (years)				
Mean ± SD	73.4 ± 6.9	69.8 ± 8.5	74.3 ± 6.1	0.021
Range	54–85	54–80	54–85	
BMI (kg/m^2^)				
Mean ± SD	26.7 ± 4.2	25.7 ± 4.7	26.9 ± 4.0	0.150
History of venous thrombosis, *n* (%)	7 (5.7 %)	3 (11.5 %)	4 (4.1 %)	0.143
Comorbidities				
Hypertension	86 (69.4 %)	20 (76.9 %)	66 (67.4 %)	0.346
Ischemic heart disease	6 (4.8 %)	0	6 (6.1 %)	0.196
Diabetes	29 (23.4 %)	7 (26.9 %)	22 (22.4 %)	0.632
Cerebrovascular disease	10 (8.1 %)	2 (7.7 %)	8 (8.2 %)	0.938
Operation time (min)				
Mean ± SD	93.7 ± 23.0	93.8 ± 21.1	93.6 ± 23.5	0.711
General anesthesia, *n* (%)	124 (100 %)	26 (100 %)	98 (100 %)	
Use of elastic stocking, *n* (%)	124 (100 %)	26 (100 %)	98 (100 %)	
Use of tourniquet, *n* (%)	124 (100 %)	26 (100 %)	98 (100 %)	
Use of cement, *n* (%)	124 (100 %)	26 (100 %)	98 (100 %)	
Prophylaxis				
Edoxaban (mg/day)	21.2 ± 7.4	21.3 ± 7.6	21.1 ± 7.4	0.891
(duration)	(11.4 ± 1.8 days)	(12.0 ± 0.6 days)	(11.3 ± 2.0 days)	0.155

*BMI* body mass index, *OA* osteoarthritis, *RA* rheumatoid arthritis

The mean age at the time of TKA was 71.0 years in the RA group and 74.3 years in the OA group. Other demographic differences between RA patients and OA patients did not reach statistical significance (Table 1). All RA patients fulfilled the 1987 American College of Rheumatology criteria for diagnosis of RA [17]. The mean disease duration was 13.3 ± 8.2 years. Twenty patients were classified in Steinbroker’s stage III and six patients in stage IV, and eight patients were classified in functional Class 2 and 18 patients in Class 3. Among 26 patients, 18 patients (69.2 %) were positive for anticitrullinated peptide antibody (anti-CCP Ab) and 15 patients (57.7 %) were positive for rheumatoid factor (RF). Among 23 patients with RA, 18 patients were treated with prednisolone (mean dose 5.0 ± 2.7 mg/day) and 10 patients were treated with methotrexate (MTX; mean dose 6.6 ± 2.8 mg/week) at the time of blood sampling.

### Risk factors for anti-PF4/heparin IgG antibody seroconversion

**Fig. 1:**
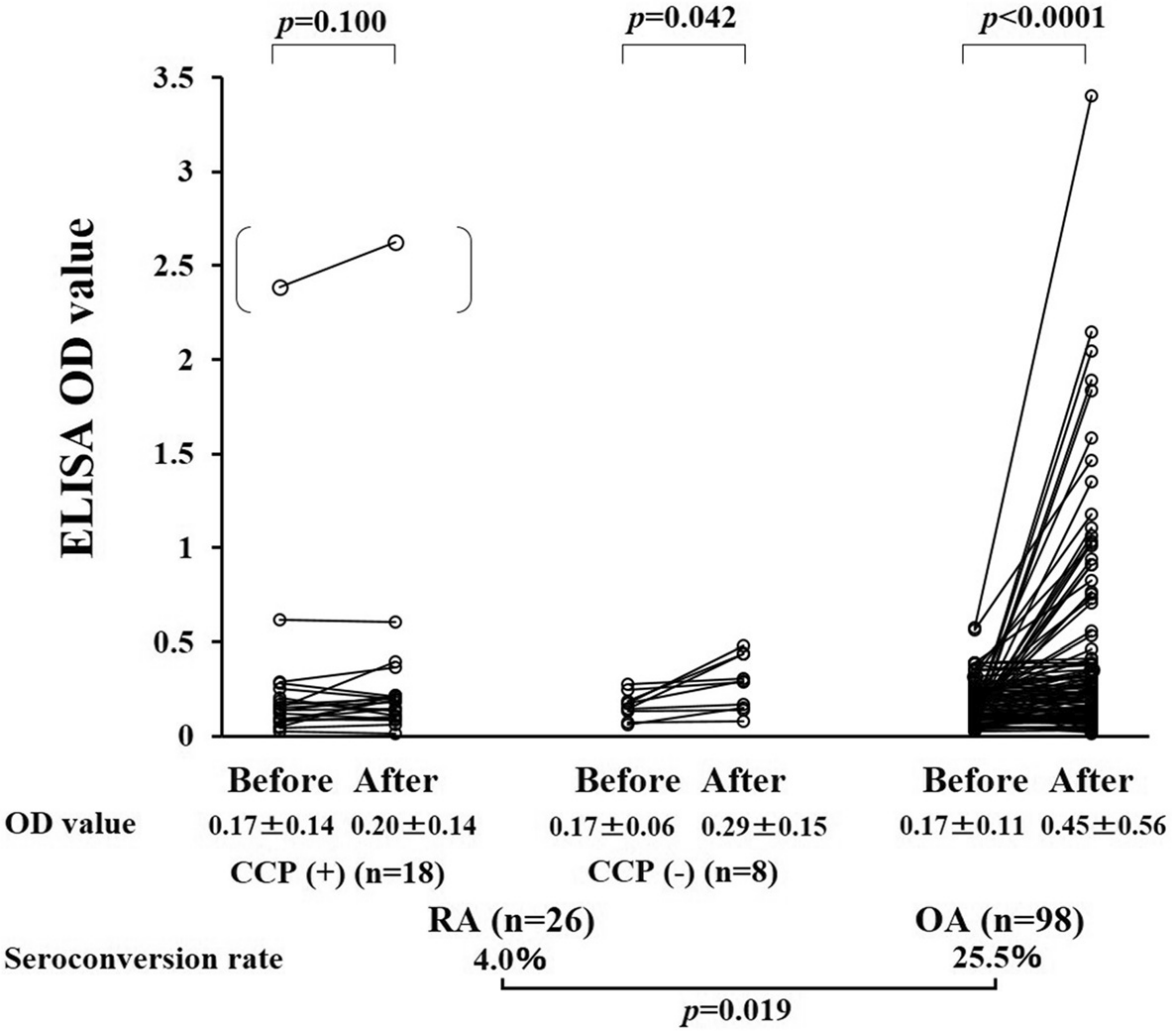
Preoperative (*before*) and POD 10 (*after*) OD levels and seroconversion rates of anti-PF4/heparin antibodies in RA patients with or without anti-CCP Ab and in OA patients. Wilcoxon signed rank test was used for the comparison of a pair of samples. One RA patient who tested positive before surgery (*dots in parentheses*) did not meet the definition of seroconversion and was excluded from the analysis. *CCP* citrullinated peptide, *ELISA* enzyme-linked immunosorbent assay, *OA* osteoarthritis, *OD* optical density, *RA* rheumatoid arthritis

Univariate analysis did not identify the variables that differed significantly between patients who did or did not become seropositive for the anti-PF4/heparin Ab postoperatively (Table 2). Next we compared the OD values for anti-PF4/heparin IgG during the preoperative and postoperative periods (Fig. 1). The seroconversion rates of anti-PF4/heparin Ab were significantly lower in RA patients than in OA patients who received TKA (4.5 % vs 25.8 %, *p* = 0.021). There was no significant difference in the anti-PF4/heparin IgG OD values before surgery and at POD 10 in RA patients with anti-CCP Ab. However, the anti-PF4/heparin IgG OD values significantly differed between the preoperative and postoperative measurements in OA patients (Fig. 1). Although there is no statistical significance, the titers of RF were lower in RA patients with seroconverted postoperative anti-PF4/heparin compared with those without postoperative ant-PF4/heparin Ab (Table 3). The titers of anti-CCP Ab, however, were significantly lower in RA patients with postoperative ant-PF4/heparin Ab compared with those without postoperative ant-PF4/heparin Ab (Table 3).

**Table 2:** Baseline characteristics of patients with high titers of anti-PF4/heparin antibody

	Postoperative seroconversion (OD ≥ 0.4)		
	Positive	Negative	
	(*n* = 28)	(*n* = 96)	*p* value
Gender(male/female)	6/22	14/82	0.275
Age (years), mean ± SD	73.8 ± 6.6	73.3 ± 7.0	0.551
BMI (kg/m^2^), mean ± SD	27.3 ± 3.9	26.5 ± 4.2	0.532
History of venous thrombosis, *n* (%)	1 (3.6 %)	6 (6.3 %)	0.503
Comorbidities, *n* (%)			
Hypertension	18 (64.3 %)	65 (67.7 %)	0.735
Ischemic heart disease	2 (7.1 %)	4 (4.2 %)	0.409
Diabetes	9 (32.1 %)	18 (18.8 %)	0.131
Cerebrovascular disease	3 (10.7 %)	7 (7.3 %)	0.401
Operation time (min), mean ± SD	91.1 ± 20.6	94.3 ± 23.7	0.682
RA/OA	3/25	23/73	0.130
Foot pump, *n* (%)	12 (42.9 %)	47 (49.0 %)	0.569
Use of steroid, *n* (%)	2 (7.1 %)	16 (16.7 %)	0.171
Use of MTX, *n* (%)	1(3.6 %)	9 (9.4 %)	0.292

*BMI* body mass index, *OA* osteoarthritis, *OD* optical density, *RA* rheumatoid arthritis, *MTX* methotrexate, *PF4* platelet factor 4

**Table 3:** Baseline characteristics of patients with high titers (OD ≥ 0.40) of anti-PF4/heparin antibody

	Rheumatoid arthritis			Osteoarthritis		
	Postoperative seroconversion (OD ≥ 0.4)			Postoperative seroconversion (OD ≥ 0.4)		
	Positive	Negative		Positive	Negative	
	(*n* = 3)	(*n* = 23)	*p* value	(*n* = 25)	(*n* = 73)	*p* value
Gender (male/female)	0/3	2/21	0.5950	6/19	12/61	0.286
Age (years), mean ± SD	67.7 ± 9.5	70.0 ± 805	0.6289	74.6 ± 6.1	74.3 ± 6.1	0.598
Disease duration (years)	14.0 ± 10.1	13.2 ± 8.2	0.7734			
BMI (kg/m^2^)						
Mean ± SD	25.2 ± 1.0	25.8 ± 5.0	0.9360	27.5 ± 4.0	26.7 ± 4.0	0.628
History of venous thrombosis, *n* (%)	1 (33.3 %)	2 (8.7 %)	0.2090	0	4 (5.5 %)	0.301
Comorbidities						
Hypertension	2 (66.7 %)	18 (78.3 %)	0.6539	17 (68.0 %)	49 (67.1 %)	0.936
Ischemic heart disease	0	0		2 (8.0 %)	4 (5.5 %)	0.482
Diabetes	1 (33.3 %)	6 (26.1 %)	0.7901	8 (32.0 %)	14 (19.2 %)	0.185
Cerebrovascular disease	1 (33.3 %)	1 (4.3 %)	0.0764	2 (8.0 %)	6 (8.2 %)	0.669
Operation time (min)						
Mean ± SD	88.0 ± 4.4	94.6 ± 22.4	0.8096	91.4 ± 21.8	94.4 ± 24.2	0.699
RF (titer IU/ml)	1/3 (17.3 ± 24.8)	14/23 (59.8 ± 73.7)	0.5558 0.1366	N/a	N/a	
Anti-CCP Ab (titer U/ml)	0/3 (0.6 ± 0.0)	18/23 (246.2 ± 560.1)	0.0215 0.0209	N/a	N/a	
CRP (mg/dl)	1.51 ± 1.09	1.24 ± 1.37	0.3086	N/a	N/a	
Use of steroid	2 (66.7 %)	16 (69.6 %)	0.9185	N/a	N/a	
Use of MTX	1 (33.3 %)	9 (39.1 %)	0.8461	N/a	N/a	
Foot pump	2 (66.7 %)	11 (47.8 %)	0.5393	10 (40.0 %)	36 (49.3 %)	0.421

*anti-CCP Ab* anti-citrullinated peptide antibody, *BMI* body mass index, *CRP* C-reactive protein, *MTX* methotrexate, *N/a* not applicable, *OD* optical density, *PF4* platelet factor 4, *RF* rheumatoid factor

### Comparison of anti-PF4/heparin antibody seroconversion rates between patients with RA and OA using the J-PSVT database

**Fig. 2:**
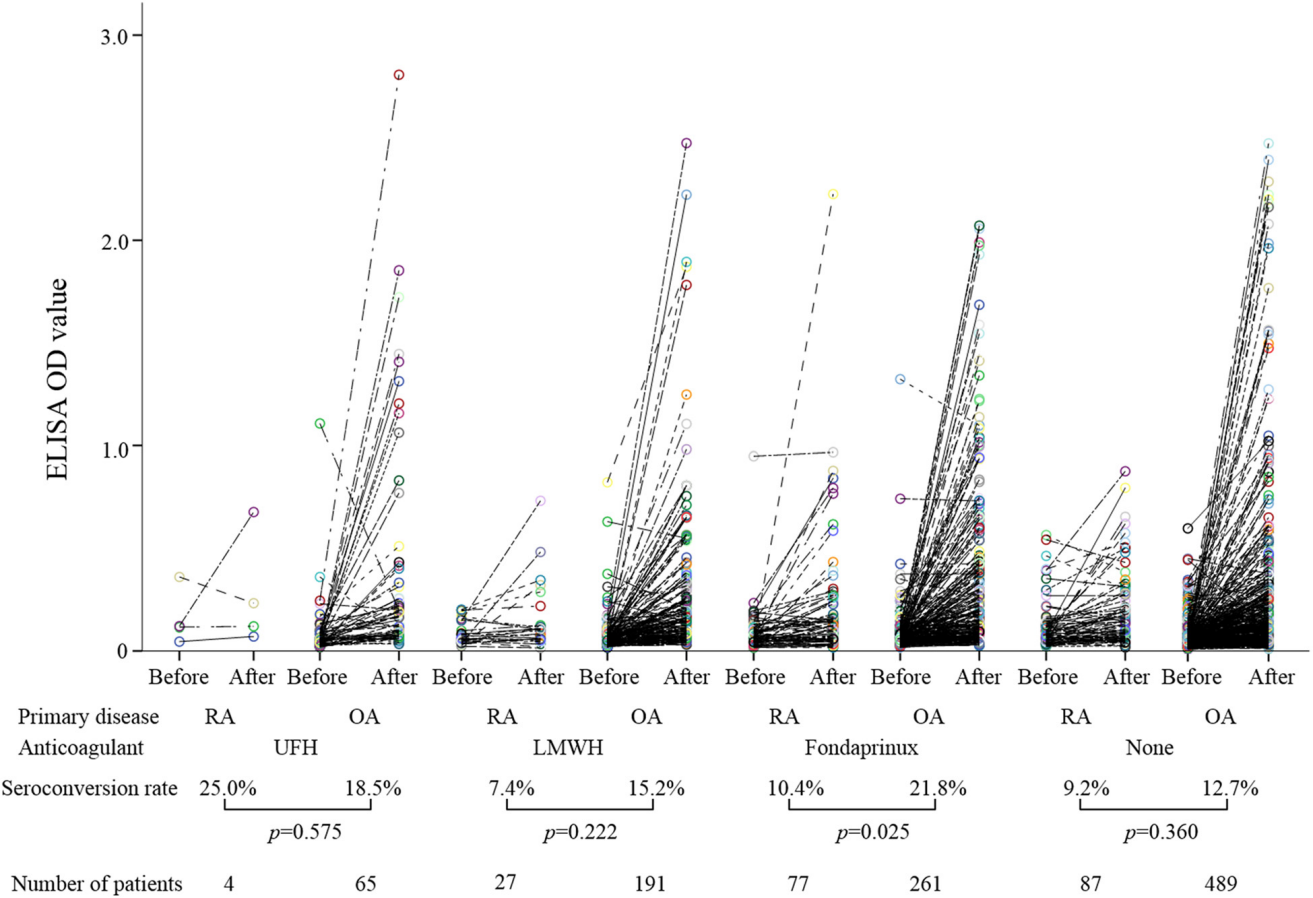
Anti-PF4/heparin seroconversion rates and proportion of patients who tested strongly positive in patients who underwent TKA. Seroconversion rate and the proportion of patients who tested positive (ELISA values ≥ 0.4 optical density (*OD*) units) were calculated for patients receiving unfractionated heparin (*UFH*), low-molecular-weight heparin (*LMWH*), fondaparinux, or only mechanical thromboprophylaxis (no anticoagulant) after TKA. Each group was divided into two subgroups based on the primary diseases (RA or OA). The seroconversion rates were compared between the RA patients or OA patients in each group using the chi-square test. When the sample size of a cell in a 2 × 2 table was less than 10, Fisher’s exact test was used instead. *p* values were not corrected for multiple hypothesis tests. *ELISA* enzyme-linked immunosorbent assay, *OA* osteoarthritis, *RA* rheumatoid arthritis

To further explore the role of primary diseases in anti-PF4/heparin Ab formation, we compared the seroconversion rates between patients with RA and with OA under various thromboprophylaxis protocols using the J-PSVT database (Fig. 2). Similar trends were observed for patients receiving LMWH or UFH; however, we were unable to demonstrate a statistically significant difference in the seroconversion rates of anti-PF4/heparin Ab between RA patients and OA patients receiving these therapies. The seroconversion rates of anti-PF4/heparin Ab, however, were significantly lower in RA patients than in OA patients who received fondaparinux. These findings are consistent with the present RCT study demonstrating that RA protects against anti-PF4/heparin Ab formation under thromboprophylaxis using a similar factor Xa inhibitor, edoxaban. We also analyzed the relationship between the seroconverting of anti-PF4/heparin Ab and the occurrence of DVT or bleeding in RA patients using this large J-PSVT database. The seroconversion of anti-PF4/heparin Ab did not affect the occurrences of postoperative DVT (anti-PF4/heparin Ab +21.1 % vs anti-PF4/heparin Ab –23.6 %) or bleeding (anti-PF4/heparin Ab +5.3 % vs anti-PF4/heparin Ab –5.6 %) in RA patients.

### Binding affinity of PF4 for heparin in RA patients and OA patients

**Fig. 3:**
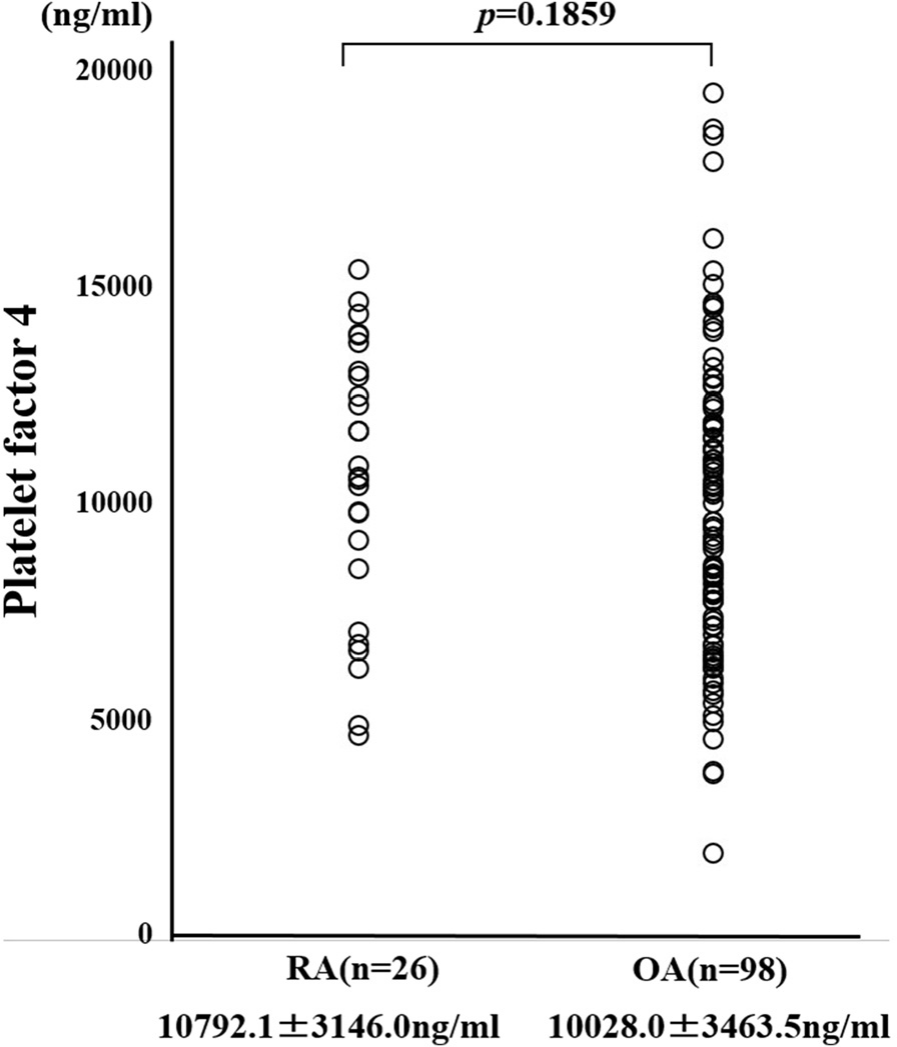
Preoperative serum levels of PF4 in RA patients and OA patients. Preoperative serum levels of PF4 were determined by ELISA in RA patients and OA patients. Results expressed as the mean ± standard deviation. There was no significant difference in serum levels of PF4 between RA patients and OA patients. *OA* osteoarthritis, *RA* rheumatoid arthritis

**Fig. 4:**
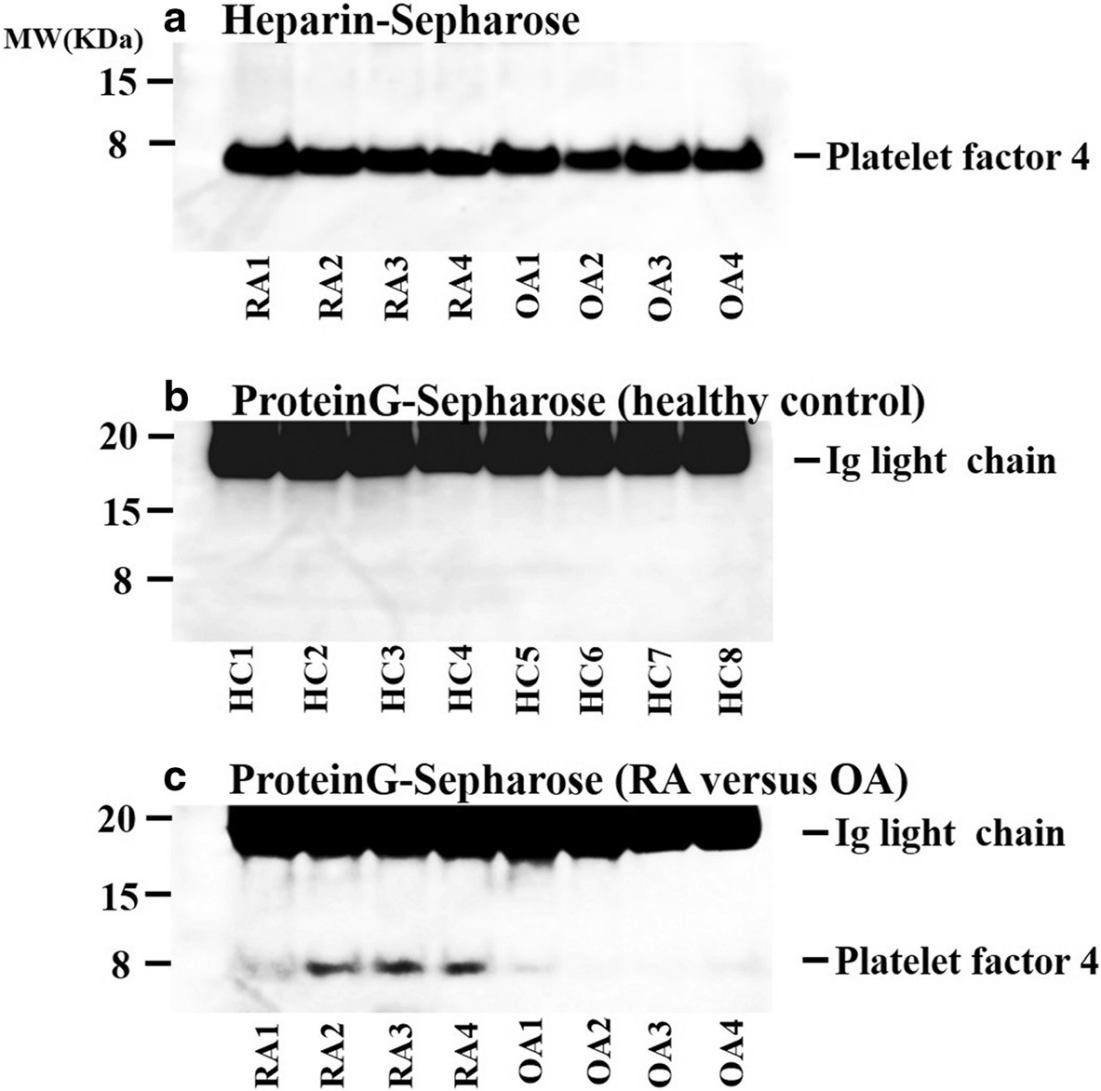
**a** Anti-PF4 immunoblot analysis using the heparin-binding fractions isolated from RA or OA patient sera. Heparin-binding fractions isolated from RA and OA patient sera using heparin-Sepharose-4B beads were subjected to anti-PF4 immunoblot analysis. PF4 bands (MW = 7800) were detected in the heparin-binding fractions from RA patients or OA patients under reducing conditions. A representative result of three independent experiments. **b**, **c** Anti-PF4 immunoblot analysis using the IgG fractions from healthy subjects (**b**) or RA and OA patient sera (**c**) using protein G sepharose-4B beads were subjected to anti-PF4 immunoblot analysis. PF4 bands (MW = 7800) were detected in the IgG fractions from RA patients under reducing conditions. Additional brood bands observed under reducing conditions are light chains generated from cleavage of the thiol-disulfide bridge of immunoglobulins. In Fig. 3c, among four patients, anti-CCP Ab plus RF were detected in two patients (RA2 and RA4). A representative result of three independent experiments. *OA* osteoarthritis, *RA* rheumatoid arthritis

We measured preoperative PF4 using the ELISA method. There was no significant difference in preoperative PF4 levels between RA patients and OA patients (Fig. 3). PF4 has been shown to bind to heparin and we hypothesized that the ability of circulating PF4 to bind heparin may differ between RA patients and OA patients. To test this, we compared the heparin-binding affinity of PF4 in RA patients and OA patients using serum samples collected prior to surgery. We used heparin-Sepharose beads to isolate PF4 with heparin-binding ability from the serum. Samples of serum from RA patients or OA patients were incubated with heparin-Sepharose beads and gently mixed. The beads were then washed and the heparin-bound materials were eluted and subjected to anti-PF4 immunoblot analysis. As shown in Fig. 4a, the bands representing heparin-bound PF4 did not differ between RA patients and OA patients, suggesting that circulating PF4 had similar heparin-binding affinity in both RA patients and OA patients.

### Circulating IgG bound to PF4 between RA patients and OA patients

Next we examined the circulating IgG bound to PF4 using sera from RA patients and OA patients. Serum samples were incubated with protein-G-sepharose beads and IgG fractions were eluted with LDS sample buffer and subjected to anti-PF4 immunoblot analysis. As shown in Fig. 4b, c, the IgG fractions isolated from healthy subjects or OA patients barely showed a PF4 band. In contrast, PF4 bands were clearly demonstrated from the IgG fractions isolated from RA patients; this suggests that the circulating IgG was bound to PF4 in RA patients. Using the sera from all RA patients (*n* = 26) and OA patients (*n* = 97), we performed the same analysis (Table 4). The PF4 bands were more frequently observed in RA patients than in OA patients (RA 18/26, 69.2 % vs OA 10/98, 10.2 %, *p* = 0.0001). In RA patients, the use of prednisolone or methotrexate did not associate with the detection of PF4 in the IgG fractions (data not shown). These results suggest that circulating IgG bound to PF4 or formed immune complexes with PF4 in the majority of RA patients.

**Table 4:** Number of patients carrying IgG-associated PF4

RA	OA	Healthy controls
(*n* = 26)	(*n* = 98)	(*n* = 13)
18/26 (69.2 %)	10/98 (10.2 %)	0/13 (0 %)

*PF4* platelet factor 4, *RA* rheumatoid arthritis, *OA* osteoarthritis

## Discussion

In this study we examined the differences in the rates of seroconversion to anti-PF4/heparin Ab positive status between patients with OA and with RA who underwent TKA. Previous studies have characterized the presence of anti-PF4/heparin Ab in patients with systemic lupus erythematosus (SLE) or anti-phospholipid Ab syndrome [5, 18]. Anti-PF4/heparin Abs have been reported to occur significantly more often in SLE patients than in healthy subjects [5]. However, the prevalence of anti-PF4/heparin Abs has not been investigated in other rheumatic diseases, including RA. To our knowledge, this is the first report documenting the frequency of seroconversion to anti-PF4/heparin Ab positive status after major joint surgery in RA patients. The most significant finding of this study was that TKA can induce seroconversion to anti-PF/heparin Ab positive status without exposure to heparin; however, in RA patients the induction of anti-PF4/heparin Ab production occurred less frequently than in OA patients. Of the OA patients who underwent TKA, 25.8 % became seropositive for anti-PF4/heparin Ab. These findings are consistent with the findings of previous studies, in which the seroconversion rates in similar patient populations that were not exposed to heparin were reported to be 14–24 % [6]. However, a significantly lower percentage of RA patients became seropositive for anti-PF4/heparin Abs after TKA than OA patients (4.5 % vs 25.8 %) in our study.

It is generally accepted that HIT is caused by antibodies which recognize complexes of PF4 and heparin [2]. The PF4 tetramer binds to heparin and glycosaminoglycans, an interaction that is central to the pathogenesis of HIT [19]. It has also been demonstrated that the binding of PF4 to certain bacteria or nucleic acids can initiate the generation of HIT-like Abs [20, 21]. Heparin has high avidity for PF4, and thus provides antigenic stimulation for HIT Abs [21]; however, a substantial number of patients who undergo major orthopedic surgery subsequently produce anti-PF4/heparin Abs without exposure to heparin. Perioperative inflammatory stimuli seem to disrupt PF4/heparin-specific B-cell tolerance [22]. In addition to the presence of heparin, the formation of immunogenic complexes that provoke HIT Abs depends on the availability of PF4. Upon release from activated platelets, PF4 rapidly associates with heparin sulfate in endothelial cells and can be brought back into circulation [23]. The availability of PF4 is therefore influenced by acute platelet activation, and logically plays a role in the risk for generation of PF4/heparin Abs during major joint surgery. However, preoperative serum PF4 levels did not differ between patients with RA and with OA.

PF4 binds to heparin-like molecules and PF4/heparin complexes stimulate anti-PF4/heparin Abs [24]. Thus, we reasoned that the increase in anti-PF4/heparin Abs after TKA might be part of the immune activation that occurs during postoperative inflammatory processes. In support of this hypothesis, we observed the appearance of anti-PF4/heparin Abs during the postoperative period after arthroplasty. However, the B-cell-mediated immune responses to PF4/heparin in RA patients were shown to be lower compared with those in OA patients. Assuming a causal relationship, we hypothesized that prescribed immunosuppressive medications, such as steroids or methotrexate, might reduce the likelihood of seroconversion to anti-PF4/heparin Ab positive status in RA patients. However, our results indicate that these medications did not affect anti-PF4/heparin Ab induction. Additionally, our data suggest that the heparin-binding affinity of circulating PF4 was not different between RA patients and OA patients.

More recently, Ohyama et al. [25] demonstrated that immune complexes containing PF4 are present specifically in the sera of RA patients, and these immune complexes could be a biomarker for the diagnosis of RA. These findings suggest that circulating PF4 is bound to IgG in established RA patients, which may contribute to the reduced likelihood of anti-PF4/heparin Abs in these patients. Although there is no statistical significance, the titers of RF were lower in RA patients with postoperative ant-PF4/heparin Ab compared with those without postoperative ant-PF4/heparin Ab. Furthermore, titers of anti-CCP Ab were significantly lower in RA patients with postoperative ant-PF4/heparin Ab compared with those without postoperative ant-PF4/heparin Ab. Immune complexes containing PF4 were found in the serum of 52 % of RA patients with anti-CCP Ab [26, 27]. It is possible that RA patients with high titers of RF or anti-CCP Ab could be preimmunized with PF4 which may be related to the abortive induction of anti-PF4/heparin Ab [27]. Our findings indicate that the circulating PF4 isolated from RA patients had a greater ability to bind IgG than the circulating PF4 in OA patients, which suggests preexisting immune responses against PF4 during the chronic immune-mediated inflammatory processes of RA. These responses may interfere with anti-PF4/heparin plasma cell differentiation through either regulatory or immune-tolerance mechanisms. The affinity of circulating PF4 for IgG in RA patients suggests that the immature B-cell immune response against PF4 occurs more frequently in RA patients than in OA patients. Our results suggest that certain immune reactions against PF4 are preexisting in RA patients. These preexisting immune responses may explain the reduced postoperative production of anti-PF4/heparin Abs in RA patients. Previous studies have demonstrated that an encounter between an immature B cell and its cognate antigen can lead to B-cell anergy [28]. This suggests a possible mechanism of B-cell tolerance for PF4/heparin complexes in patients with chronic inflammatory disorders such as RA. A recent study of anti-PF4/ heparin immunization in patients after cardiac surgery suggested that perioperative inflammation affects the immune response [29]. Alternatively, chronic rheumatoid inflammation may downregulate these perioperative inflammations that contribute to occurrence of anti-PF4/ heparin Ab in RA patients.

This study had several limitations. One major limitation was the potential for several biases in this quasi-randomized controlled trial. We were not able to obtain sufficient numbers of patients (particularly RA patients) to achieve good statistical power, so there is the undeniable possibility that the study may have been underpowered. Also, the J-PSVT does not provide information related to the RA severity score or data regarding treatments, such as antirheumatic drugs and glucocorticosteroids, so we were not able to adjust for these in our statistical models.

## Conclusions

This study revealed a lower rate of seroconversion to anti-PF4/heparin IgG positive status after TKA in RA patients than in OA patients. This suggests that the autoimmune-like features of anti-PF4/heparin Ab production cannot be explained by the postoperative induction of this antibody. Our data provide further evidence for the unique profile of anti-PF4/heparin Ab induced during the postarthroplasty phase without exposure of heparin.

## Abbreviations

DVT: Deep vein thrombosis
HIT: Heparin-induced thrombocytopenia
J-PSVT: Japanese Study of Prevention and Actual Situation of Venous Thromboembolism after Total Arthroplasty
LMWH: Low-molecular-weight heparin
OA: Osteoarthritis
OD: Optical density
PF4: Platelet factor 4
POD: Postoperative day
RA: Rheumatoid arthritis
SLE: Systemic lupus erythematosus
TKA: Total knee arthroplasty
UFH: Unfractionated heparin
VTE: Venous thromboembolism
